# The influence of older age, individual differences in cognitive abilities, and state of mind on learning novel categories

**DOI:** 10.1007/s00426-026-02334-1

**Published:** 2026-06-25

**Authors:** Kana Kimura, Madeline R. Valdez, Caitlin R. Bowman

**Affiliations:** 1 Department of Psychological and Brain Sciences, University of Wisconsin-Milwaukee, Milwaukee, USA; 2 Department of Psychological and Brain Sciences, Washington University in St. Louis, St. Louis, USA

## Abstract

**Objectives:**

Category learning involves learning group items based on similar feature values, and it is a key ability that allows us to acquire new concepts and skills throughout our lives. Yet we know little about the traits and situational factors that facilitate category learning and how those differ in older age. Here, we test the relationship between categorization performance and individual differences in a variety of cognitive abilities and aspects of state of mind and how those relationships differ in older age.

**Method:**

Seventy-six young adults (18–29 years old) and 73 older adults (60–83 years old) underwent a cognitive assessment testing processing speed, working memory, verbal comprehension, and perceptual reasoning. Two categorization sessions followed where participants completed a categorization task and state of mind questionnaires assessing stress, motivation, mood, and sleep.

**Results:**

An individual’s cognitive abilities were significant positive predictors of categorization test accuracy. This effect was not moderated by age groups. Most state of mind variables were not significant predictors of categorization test accuracy, except that young adults with negative mood performed worse on the categorization test compared to older adults.

**Discussion:**

We show that baseline cognitive abilities influence categorization test accuracy more than state of mind variables regardless of age. Thus, interventions may be most effective when they are designed to work with individual’s existing knowledge rather than attempt to directly manipulate transient states of mind.

## Introduction

Category learning is a key cognitive ability that involves learning to relate items to one another based on their shared qualities, often giving them a common label. The ability to learn new categories is relevant throughout the lifespan, like when we pick up a new hobby or when a new concept emerges in the world. For example, becoming a bird watcher involves learning to identify and classify birds by their species, typically based on their visual features and vocalizations. This kind of new conceptual learning has been shown to be important for maintaining one’s overall cognitive abilities in older age, improving quality of life and reducing isolation (Park et al., 2014). Despite the importance of category learning, it has received less attention in the cognitive aging literature compared to other cognitive domains. Although most category learning studies have been conducted with young adults, aging studies of categorization have tended to show age-related impairments (Badham et al., 2017; Bowman et al., 2022; Davis et al., 2012; Filoteo & Maddox, 2004; Rabi & Minda, 2016; Wahlheim et al., 2016). Thus, understanding the factors that can promote successful category learning in older adults has the potential to alleviate cognitive deficits and enhance quality of life.

We know from studies in young adults that category learning is a multidimensional cognitive ability involving perception, attention, memory, language, and decision-making (Lewandowsky et al., 2002; Markman & Ross, 2003; Rehder & Hoffman, 2005; Shepard et al., 1961; Zeithamova & Maddox, 2007). Therefore, individual differences in these abilities may contribute to the variability in category learning performance. Most broadly, poor categorization abilities could be a sign of cognitive impairment. Indeed, one prior study showed that older adults with cognitive impairment performed poorly in categorization tasks (Romero-Ayuso et al., 2021). However, it is not known whether more subtle differences in cognitive health within a sample of older adults who meet the ‘healthy aging’ threshold also relate to categorization abilities.

Further, abilities within specific cognitive domains may be particularly important for maintaining categorization abilities in older age. Knowing which cognitive abilities are most related to the ability to learn new concepts could help identify older adults who are likely to have difficulty learning new categories, which could make those individuals more likely to withdraw from the types of cognitive engagement that would help them preserve the abilities that they have. One study, for example, argues that verbal abilities are especially critical for category learning (Lupyan et al., 2007). In their work, participants were asked to categorize whether “aliens” are approachable or to be avoided, and participants who learned categories with labels showed more robust category learning than those who learned without labels. Studies of healthy aging tend to show relatively modest declines in verbal knowledge (Salthouse, 1993), leaving open the possibility that older adults rely on verbal abilities to support category learning. Yet, given that category learning abilities tend to decline in older age while verbal knowledge is more stable, verbal abilities alone do not seem to be enough to maintain categorization performance or cannot do so for all older adults.

Fluid intelligence abilities like working memory (Baddeley, 1992) and processing speed (Salthouse, 1993) are known predictors of categorization abilities in young adults (Lewandowsky, 2011; Lewandowsky et al., 2012). These findings suggest that successful category learning draws heavily on the capacity to rapidly encode stimulus features, maintain them across trials, and update internal representations as feedback is received. Categories are thought to be optimally learned by selectively attending to relevant features and maintaining them in working memory from trial-to-trial (Ashby et al., 1998; Nomura et al., 2007; Nosofsky, 1986). Both working memory (Light & Anderson, 1985; Salthouse & Babcock, 1991) and processing speed (Kail, 1986; Salthouse, 1996) are known to decline in older age, which may make these abilities particularly relevant for older adults’ categorization abilities. Further, these abilities may be especially relevant when to-be-categorized stimuli contain many relevant dimensions and with time pressure to process them. Categorization performance may also relate to perceptual reasoning abilities, which involve detecting and completing perceptual patterns and relationships (Goldstone, 1998). Visual category learning typically involves detecting visual features that are shared among members of the same category (Ashby & Maddox, 2005; Richler & Palmeri, 2014; Sloutsky & Fisher, 2004), and it may be that some individuals are more skilled than others at detecting these commonalities. Some studies suggest that perceptual reasoning abilities decline with age (Abbenhuis et al., 1990; Small et al., 1995), but Scheiber et al. (2017) show that 70–80% of the variance in the declined perceptual reasoning ability is accounted for by slowed processing speed in older adults. Taken together, the extent to which individual differences in cognitive abilities track categorization performance and whether any such relationships differ with age is an open question.

Looking beyond relatively stable cognitive abilities, individuals also vary in their cognitive performance from day-to-day based on their state of mind. This kind of intraindividual variability is especially important to consider in aging because intraindividual variability tends to increase in older age in ways that relate to cognitive performance (Bielak et al., 2014; Martin & Hofer, 2004; Prigatano et al., 2025; Roalf et al., 2018). Stress (de Kloet et al., 1999; Sandi, 2013), mood (Bower et al., 1983; Forgas, 2008), motivation (Braver et al., 2014.; Donovan, 2015), and sleepiness (Diekelmann & Born, 2010; Fortier-Brochu et al., 2012; Jones & Harrison, 2001) are all aspects of state of mind that influence young adults’ performance in other cognitive domains. Yet their impact on category learning and whether older age moderates any impact is not known.

The limited work on the influence of state of mind on categorization performance in young adults provides some evidence that it may be an important factor affecting learning and generalization. Acute, laboratory-induced stress enhanced learning in young adults across several different category structures (Ell et al., 2011; McCoy et al., 2014), and stress responses associated with a subset of category members during learning can generalize across the category (Dunsmoor & Murphy, 2015). Greater subjective stress and stressful life events are associated with steeper age-related declines in episodic memory (VonDras et al., 2005). It remains unclear, however, whether similar patterns extend to category learning. Everyday life stress in older adults could impair category learning, but it is also possible that it follows the similar patterns to the laboratory findings in young adults and enhances category learning. Better mood is also associated with better category learning in young adults (Nadler et al., 2010), which may be because positive mood enhances cognitive flexibility (Ashby et al., 1999). Yet it is unknown how these mood effects influence older adults’ ability to learn new categories. Motivation has been related to long-term memory in young adults (Cook et al., 2015; Miendlarzewska et al., 2016; Murayama & Elliot, 2011; Murty & Dickerson, 2017), but it is unclear if category learning performance shows a similar sensitivity to motivation. Older adults’ cognitive and memory performance tends to increase as the personal relevance to the cognitive task increases (Germain & Hess, 2007; Hess et al., 2001; Hess, 2005), which may mean that motivation to learn a new category may be a particularly important predictor of older adults’ performance. Lastly, poor sleep quality and current sleepiness are associated with poor performance across many cognitive domains (Diekelmann & Born, 2010; Elhami Athar et al., 2020; Fortier-Brochu et al., 2012; Jones & Harrison, 2001; Nebes et al., 2009; Smith et al., 2002; Steenari et al., 2003; Stickgold, 2005; Telzer et al., 2013). Sleep may be particularly relevant for older adults because decreases in amount and quality of sleep are common in older age (Blay et al., 2008). Sleep disturbances are known to impact episodic memory in older adults (for review, see Yeh et al., 2018), but the impact on category learning is not known.

In the present study, we explored how cognitive abilities and state of mind impact categorization performance and how older age may moderate these relationships. To do so, we used data from an existing study of categorization (Valdez, Kimura, & Bowman, in press) that recruited 94 young (18–30 years old) and 85 older (60 + years old) adults. Young adults were included as a comparison group to establish a baseline pattern of cognitive and affective predictors of categorization performance. This allowed us to determine whether any relationships identified emerged or became stronger in older age or were instead present in young adulthood as well. Because prior literature provides mixed findings, the present study was exploratory and did not specify directional age-by-predictor interactions. In the first study session, participants completed a cognitive assessment that included the Mini-Mental State Exam (Folstein et al., 1975) to evaluate overall cognitive health and screen for signs of cognitive impairment, and portions of the Weschler Adult Intelligence Scale – IV (Wechsler, 2008) to get more fine-grained measures in several cognitive domains. In the second and third sessions (approximately 48 h apart), participants completed category learning and generalization tasks. In each session, participants learned to sort novel cartoon animals into two categories that were each organized around a prototype that represented the typical features of all category members (Posner & Mitchell, 1967). Prior work has shown that, while some age deficits are present in prototype-based categories, older adults can nonetheless learn them and sometimes generalize category labels to new examples with comparable accuracy to young adults (Bowman et al., 2022; Valdez, Kimura, & Bowman, in press). In each categorization session, participants also completed a battery of questionnaires assessing their state of mind in terms of stress, mood, motivation, and sleepiness. Because prior findings in this area are mixed and no strong theoretical predictions can be derived, the present study was conducted as an exploratory investigation. In the present study, we aimed to investigate age differences in (1) the relationship between cognitive abilities and categorization performance and (2) the relationship between intraindividual differences in state of mind and categorization performance.

## Materials and methods

### Data Availability

The current study was not pre-registered. The data and analysis code for the present study are freely available through the Open Science Framework (https://osf.io/gsn47/). The stimuli are also freely available (https://osf.io/8bph2/). A separate manuscript investigating age differences in the stability of categorization strategies with the same dataset is published (Valdez, Kimura & Bowman, in press).

### Participants

All participants completed written informed consent prior to completing the experiment and the experimental protocol was approved by UW-Milwaukee’s Human Research Protection Program (#21.300). An a priori power analysis determined that *n* = 80 participants per age group were needed to detect an individual difference relationship of *|r|* > 0.3 in each age group with alpha = 0.05 and 80% power. To reach that sample size, a total of 94 cognitively healthy young adults from the University of Wisconsin-Milwaukee and 85 cognitively healthy older adults from the surrounding Milwaukee area were recruited to participate in this study for course credit or monetary compensation. Nine young adults and two older adults were excluded because they did not complete all sessions or did not come back for the second session within a week. Three older adults were additionally excluded for failure to follow task instructions. Data from nine young adults and six older adults were lost after data collection was complete when the data storage server was reconfigured. One older adult did not pass our criteria of being cognitively healthy (Mini Mental State Exam score < 25). After exclusions, a total of 76 young adults (mean age = 21.4, SD = 2.6, age range = 18–29, mean years of education = 14.5, SD = 1.5) and 73 older adults (mean age = 70.0, SD = 5.9 years, age range = 60–86, mean years of education = 16.1, SD = 2.6) were included in the analyses. Although our final sample was smaller than we anticipated, a sensitivity analysis revealed that it is still sufficient to detect *|r|* > 0.32 within each age group. Demographic information for included participants is provided in Table 1.

**Table 1:** Demographic characteristics of participants

	Young adults *N* = 76		Older adults *N* = 73	
	*n*	%	*n*	%
Gender				
Female	57	75	36	49
Male	16	21	30	41
Non-binary	2	3	0	0
Transgender	1	1	0	0
Prefer not to answer	0	0	7	10
Race				
White	49	64	66	90
Black/African American	4	5	2	3
Hispanic/Latino	3	4	0	0
Asian	7	9	0	0
Hawaiian/Pacific Islander	0	0	1	1
Multiracial	8	11	0	0
Prefer not to answer	5	7	4	5

All participants completed the Mini Mental State Exam (MMSE; Folstein et al., 1975) prior to the categorization tasks to assess cognitive health. Participants who scored > 24 were considered cognitively healthy and were eligible to continue with the tasks. MMSE scores between young (M = 28.7, SD = 1.7) and older adults (M = 28.5, SD = 1.4) did not differ significantly (*t*(147) = 0.79, *d* = 0.19, *p* = .43). Neuropsychological testing also included 8 subtests of the Wechsler Adult Intelligence Scale IV (Wechsler, 2008). Scores from the WAIS subtests were organized around four theoretically and empirically supported cognitive domains: Verbal Comprehension Index (VCI; comprised of Vocabulary and Information), Perceptual Reasoning Index (PRI; comprised of Matrix reasoning and Visual puzzles), Working Memory Index (WMI; comprised of Digit span and Arithmetic), and Processing Speed Index (PSI; comprised of Symbol search and Coding). The use of these composite scores follows the facet structure of the instrument and aligns with standard interpretive practice.

Mean scores for WAIS full-scale IQ, four WAIS indices, and each subtest are reported in Table 2. We report two types of scores: the age-corrected (scaled) scores and a composite score that did not adjust based on age norms (age-uncorrected scores). We included the age-corrected scores to give a sense of how cognitively representative our samples were of the larger population, keeping in mind that they were screened to be cognitively healthy which is likely to bias the distribution toward higher scores. WAIS-IV age corrected scores are created by converting raw subset scores into scaled scores (M = 10, SD = 3) provided in the WAIS-IV manual. These scaled scores are then combined into index scores and full-score IQ with mean of 100 and standard deviation of 15. Because the standard WAIS-IV scaling procedure is designed to compare individuals to others in a similar age range, it (intentionally) removes most age-related variability in scores that was of interest for our study. We thus included age-uncorrected scores as primary predictors of interest in analyses linking cognitive abilities to categorization test accuracy. Because subtests that comprise a given composite score are on different scales (e.g., Information is out of 24 points and Vocabulary is out of 55 points, but both are included in Verbal Comprehension), we z-scored each raw subtest score across the full sample (regardless of age) before averaging across subtests that were part of the same index (e.g., verbal comprehension, VCI). We performed this conversion of raw scores to place all the scores on a comparable scale and allow us to examine individual differences without the built-in age adjustments that could otherwise hinder detection of age effects. In our study, older adults showed higher age-uncorrected scores on full-scale IQ, VCI, and WMI compared to young adults, but young adults outperformed older adults on PRI and PSI (all *t*’s > 2.0, *p*’s < 0.05, *d*’s > 0.33). Both group means were within one standard deviation of the population mean on each age-corrected WAIS score.

**Table 2:** WAIS-IV test scores

Measure	Age-corrected scores			Age-uncorrected scores		
	YA	OA	*p*	YA	OA	*p*
Full-scale IQ	101.3 (11.8)	109.9 (12.5)	< 0.001			
VCI	107.9 (14.1)	113.4 (14.0)	0.02	− 0.76 (1.68)	0.79 (1.62)	< 0.001
Information	10.71 (2.83)	11.82 (2.38)	0.01	− 0.37 (0.94)	0.39 (0.91)	< 0.001
Vocabulary	11.85 (2.57)	12.88 (2.99)	0.03	− 0.38 (0.92)	0.40 (0.93)	< 0.001
PRI	102.4 (13.5)	110.4 (14.6)	< 0.001	0.69 (1.60)	− 0.72 (1.63)	< 0.001
Visual puzzle	10.72 (2.69)	11.48 (3.16)	0.12	0.44 (0.92)	− 0.46 (0.86)	< 0.001
Matrix reasoning	10.03 (2.80)	12.19 (2.69)	< 0.001	0.25 (0.95)	− 0.26 (0.98)	0.0014
WMI	95.5 (12.8)	106.2 (12.7)	< 0.001	− 0.28 (1.67)	0.29 (1.72)	0.04
Digit span	8.61 (2.18)	10.85 (2.50)	< 0.001	− 0.11 (0.94)	0.11 (1.05)	0.18
Arithmetic	10.08 (2.57)	11.56 (2.61)	< 0.001	− 0.17 (1.03)	0.18 (0.94)	0.03
PSI	99.7 (14.6)	108.7 (13.0)	< 0.001	0.60 (1.79)	− 0.63 (1.67)	< 0.001
Coding	9.91 (2.79)	12.30 (2.53)	< 0.001	0.23 (0.96)	− 0.24 (0.99)	0.004
Symbol search	9.92 (3.03)	10.93 (2.77)	0.03	0.38 (0.97)	− 0.39 (0.87)	< 0.001

Note. *P*-values reflect independent-samples t-tests comparing young adults (YA) and older adults (OA) on each measure. VCI = Verbal comprehension index; PRI = Perceptual reasoning index; WMI = Working memory index; PSI = Processing speed index

### Materials

#### Questionnaires

Participants completed a set of computerized questionnaires that assessed their state of mind at the beginning of each categorization session, with the order of the questionnaires counterbalanced across participants. Mean and standard deviation of scores for each state of mind variable and their age differences are shown in Table 3. Current state stress level was measured with the Short Stress State Questionnaire (Helton, 2004; Helton et al., 2005; Helton & Garland, 2006; Helton & Russel, 2010; Helton & Näswall, 2015), and we used the overall average across sub scores as an indicator of stress. Motivation was measured with the situational motivation scale (Guay et al., 2000). The overall combined score was used to determine participant’s motivation to complete our tasks. Mood was measured with the Positive and Negative Affect Schedule (PANAS; Watson et al., 1988). Both positive affect and negative affect were separately calculated using PANAS manuals. Sleep quality, duration of sleep, and subjective sleepiness were measured by Stanford sleepiness scale (Hoddes et al., 1973) and the St. Mary’s hospital sleep questionnaire (Ellis et al., 1981). For sleep quality, we used the sum of the score of the sleep quality of the night before the session and hours of sleep from the St. Mary’s hospital sleep questionnaire. The raw score of the participant’s answer to current sleepiness in the single-item Stanford sleepiness scale was used to assess participant’s current sleepiness.

**Table 3:** Experiment scores by session

Measurements	Session 1			Session2		
	YA	OA	*p*	YA	OA	*p*
Stress	2.57 (0.43)	2.35(0.34)	0.001	2.47 (0.46)	2.37 (0.36)	0.15
Positive affect	27.7 (8.0)	35.0 (8.0)	< 0.001	27.1 (8.0)	34.6 (8.7)	< 0.001
Negative affect	15.5 (5.4)	11.6 (2.7)	< 0.001	14.9 (5.6)	11.5 (3.2)	< 0.001
Motivation	69.8 (10.8)	69.3 (12.8)	0.78	67.0 (10.8)	67.7 (13.3)	0.70
Sleep quality	12.3 (2.6)	12.4 (2.3)	0.72	12.5 (2.5)	12.5 (2.3)	0.97
Current sleepiness	3.7 (1.8)	3.1 (2.0)	0.04	3.7 (1.7)	3.0 (1.8)	0.02
Categorization	0.73 (0.12)	0.68 (0.14)	0.02	0.75 (0.13)	0.70 (0.13)	0.02

*Note. P*-values reflect independent-samples t-tests comparing young adults (YA) and older adults (OA) on each measure

#### Categorization stimuli

**Fig. 1:**
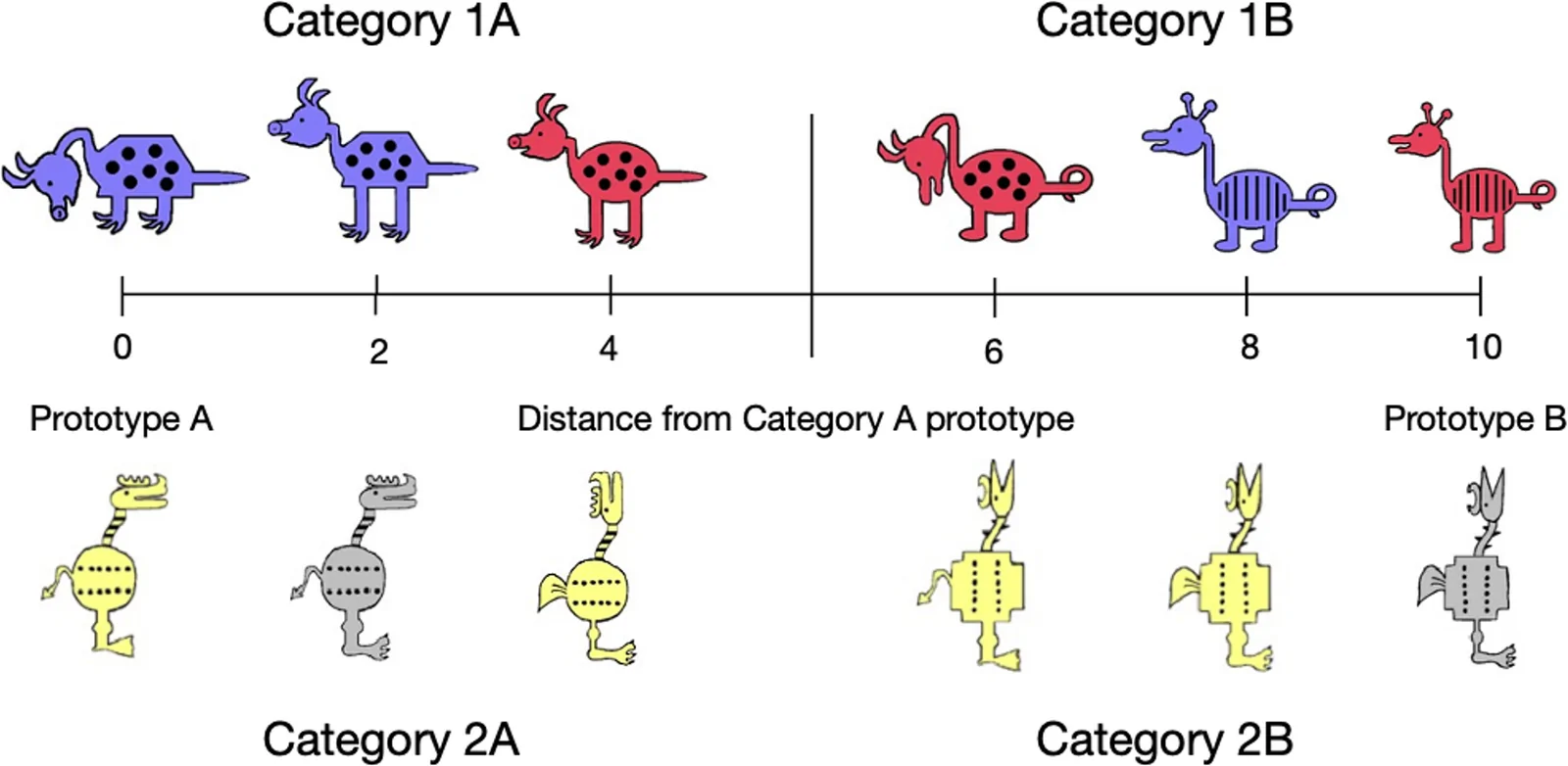
Example stimuli and category structure

Stimuli consisted of two sets of cartoon animals that differed on 10 binary features (see Appendix for description of features; Bozoki et al., 2006; Zeithamova & Bowman, 2020; stimuli available for download at https://osf.io/8bph2/). For each participant, one stimulus set was assigned as the stimulus set for the first categorization session, and the other set was assigned to the second categorization session. The stimulus set assignment was counterbalanced across participants. Figure 1 depicts the general structure of the categories. For each participant, the prototype for Category A was randomly selected from two possible stimuli. The item that shared no features with the Category A prototype was assigned as the prototype for Category B. Physical distance was defined as the number of features an item differed from the Category A prototype, and category membership was determined by the number of features that an item shared with the category prototypes. A stimulus was considered a member of Category A if it shared more features with the Category A prototype than the Category B prototype, and vice versa for Category B membership. The category learning task was identical in structure across sessions, but the cartoon animals and category names were different between sessions. The group names were “Batmans” and “Robins” for one session, and “Wallaces” and “Gromets” for the other session. The group names were counterbalanced across sessions.

##### **Training set**

Table 4 depicts the training set structure. Each training set consisted of 7 items from each category (14 total across the two categories): four items at Distance 2 and three items at Distance 4 from the category prototypes. The training items were selected so that all features were equally predictive of category membership, and they were re-coded based on the randomly selected prototype.

##### **Generalization test set**

Both training items and new items were shown during the test phase. Both prototypes and 10 new stimuli at each distance (1–4, 6–9) were added during the test. Items equidistant from the category prototypes (distance 5) were not included in the test set. In total, 96 unique items were included in the test set.

**Table 4:** Dimension values of example prototypes and training stimuli from each category

Dimension values										
Stimulus	1	2	3	4	5	6	7	8	9	10
Prototype A	1	1	1	1	1	1	1	1	1	1
A1	1	0	1	1	1	1	1	1	0	1
A2	1	1	1	0	1	1	0	1	1	1
A3	1	1	0	1	1	1	1	0	1	1
A4	0	1	1	1	1	1	1	1	1	0
A5	1	1	1	0	0	0	0	1	1	1
A6	1	1	0	1	0	0	1	0	1	1
A7	0	0	1	1	1	1	1	1	0	0
Prototype B	0	0	0	0	0	0	0	0	0	0
B1	0	1	0	0	0	0	0	0	1	0
B2	0	0	0	1	0	0	1	0	0	0
B3	0	0	1	0	0	0	0	1	0	0
B4	1	0	0	0	0	0	0	0	0	1
B5	0	0	0	1	1	1	1	0	0	0
B6	0	0	1	0	1	1	0	1	0	0
B7	1	1	0	0	0	0	0	0	1	1

Note. Prototypes are included as references but not included in the training set

### Procedure

**Fig. 2:**
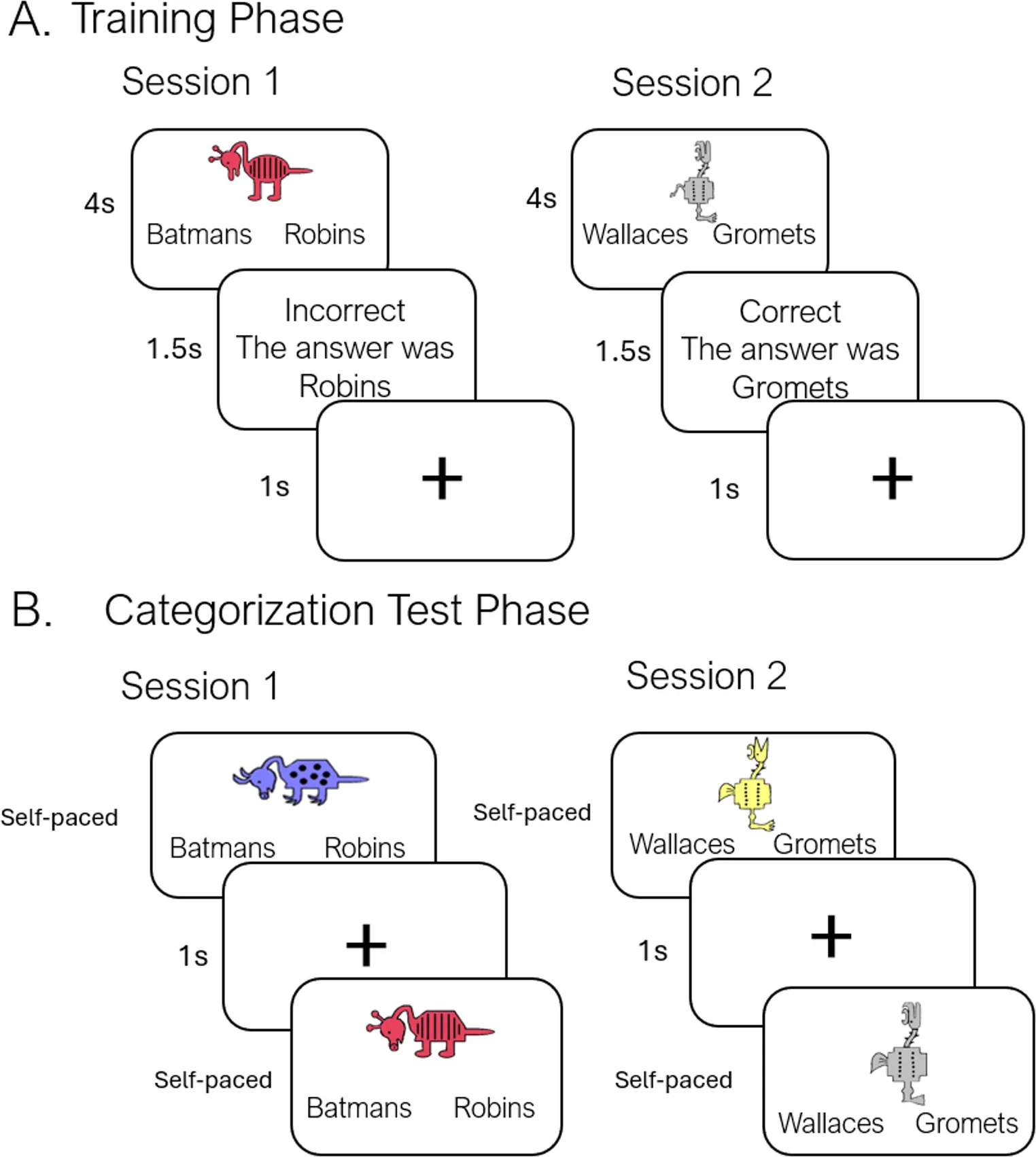
Categorization task procedures

The study consisted of three sessions: an initial cognitive assessment, followed by two separate category learning sessions. The time between the cognitive assessment session and the categorization task sessions varied (range: 2–7 days). Participants completed two category learning sessions approximately 48 h apart (SD = 18.93). One participant had a shorter delay between sessions (27 h), and 13 participants had a delay longer than three days. 91% of participants had a delay of 46–53 h. At the start of each categorization task session, participants completed the five state-of-mind questionnaires. Participants then completed the category training phase (Fig. 2A) followed by the categorization test (Fig. 2B) for each categorization task session.

On each training trial, a training item along with both category labels were presented on the screen for 4 s, and participants were instructed to pick which category they thought the animal belonged to. They respond by pressing “f” or “j” on keyboard while the stimulus was on screen. Participants were told that they would not know which group the animals belonged to at first, but that they would receive feedback that could be used to learn which group they belonged to. The stimulus and category labels remained on the screen for 4 s regardless of participants’ response to ensure each stimulus had the same amount of exposure. Corrective feedback (Correct vs. Incorrect) and the correct label (e.g., ‘The answer was Wallaces’) was given immediately after the participant’s response, replacing the response options on the screen. Feedback remained on the screen for 1.5 s after the stimulus disappeared. If participants failed to respond within 4 s, they were given a warning message that their response was too slow and were informed on what the correct category was. Each training trial was followed by a 1-second fixation cross intertrial interval. The training phase consisted of 8 training blocks separated by self-paced breaks. Each training item was shown 3 times during a single training block, for a total of 42 trials per block and 336 total trials during training. Training stimuli were shown in a random order in each block with the constraint that all items were shown once before any item was repeated.

On each categorization test trial, an item was presented along with two category labels and participants were instructed to choose which category they believed the animal belonged to. During the test phase, participants did not receive feedback. Test trials were self-paced and were followed by a 1-second fixation cross intertrial interval. The categorization test phase had two blocks separated by a self-paced break. Each block consisted of 56 trials. All training items and both category prototypes were shown twice (once per block), and 10 new stimuli at each distance (1–4, 6–9) were each shown once during the test. Test stimuli were shown in a random order for each participant, with the constraint that all training items and prototypes were shown once before any item was repeated.

Existing literature reported that time of day affects category learning (Tandoc et al., 2021), and with the nature of our study investigating the effect of sleep before the night of session on categorization test accuracy, it is important to look at the time of the day of sessions for both young and older adults. Both age groups had similar visit times, with the most frequent visits at 10am. Their time of session did not differ significantly between age groups either in session 1 (*t*(147) = 0.14, *p* = .89, *d* = 0.02) or session 2 (*t*(147) = 0.51, *p* = .61, *d* = 0.08).

### Statistical analyses

We focused on the cognitive abilities and state of mind factors that relate to overall categorization test accuracy as indexed by average accuracy across all test items. We used overall test accuracy rather than separate accuracies for trained items versus generalization items because those scores were highly correlated with one another at the individual differences level (Session 1 *r* = .77, *p* < .001; Session 2 *r* = .79, *p* < .001), limiting our ability to identify unique cognitive correlates of each separately.

Our general approach for relating cognitive abilities to categorization test accuracy was to compute multiple regressions with a given cognitive assessment score, effect-coded age group (-0.5 = young, 0.5 = older), and the cognitive score x age group interaction effect as predictors of the categorization test performance. For the state of mind variables, we used a linear mixed effects modeling approach because we had separate state of mind scores and categorization scores for each session. This allowed us to consider the effect of session when computing the state of mind-categorization relationship. Note, however, that with only two timepoints, we could only reliably estimate a random intercept, not random slopes, which limits our ability to detect true within-person state of mind effects. For each model, we included the state of mind variable, effect-coded age group (-0.5 = young, 0.5 = older), and their interaction as fixed effects. Session was also included as a fixed effect. Subjects were considered as a random effect. To quantify the evidence for the absence of effects when the null hypothesis could not be rejected, we conducted Bayesian model comparisons using the *BayesFactor* package in R (Morey & Rouder, 2011). For null predictors, we compared a model including the predictor to model excluding it. Bayes factors (BF_01_) were computed using default Cauchy priors on effect sizes. Values of BF_10_ > 1 indicate evidence in favor of the null model, with larger values reflecting stronger support for the absence of an effect. Following conventional guidelines, BF_01_ values between 1 and 3 provide anecdotal evidence for the null, values between 3 and 10 provide moderate evidence, and values over 10 indicate strong evidence for the absence of an effect (Jeffreys, 1998; Kass & Raftery, 1995). When computing multiple models on the same data, we computed a Bonferroni correction for multiple comparisons to guard against false positives in these exploratory analyses. We note the number of comparisons corrected for in each section. All the statistics were performed using the R statistical program, version 4.4.0. Linear mixed effects models were conducted using lme4 package (Bates et al., 2015).

## Results

### Categorization performance

Age-related differences in categorization performance and strategy use with a slightly different sub-sample were published in a separate manuscript (Valdez, Kimura & Bowman, in press), which we describe briefly to provide context for the present results without duplicating analyses. In summary, younger adults had higher training accuracy than older adults, especially for items further from the category prototypes. Training accuracy was relatively stable across sessions. Across both sessions, consistent with their advantage during training, young adults had higher test accuracy than older adults for the trained items, but age differences were not significant for generalization items. To avoid duplicate reporting and maintain conceptual clarity between manuscripts, the present study reports overall categorization accuracy as the primary outcome measure. Overall categorization test accuracy for each session is presented in Table 3. In both session 1 and session 2, young adults outperformed older adults in categorization test accuracy (all |*t*’s| > 2.34, *d*’s > 0.38, *p*’s < 0.03). Thus, we found evidence of an age-related deficit in categorization, likely driven primarily by age deficits in memory for trained items and less so by deficits in generalization. For the subsequent analyses, the patterns of findings were very similar when we separated old (trained) vs. new items for categorization vs. generalization, respectively.

### Intercorrelation of cognitive abilities

**Fig. 3:**
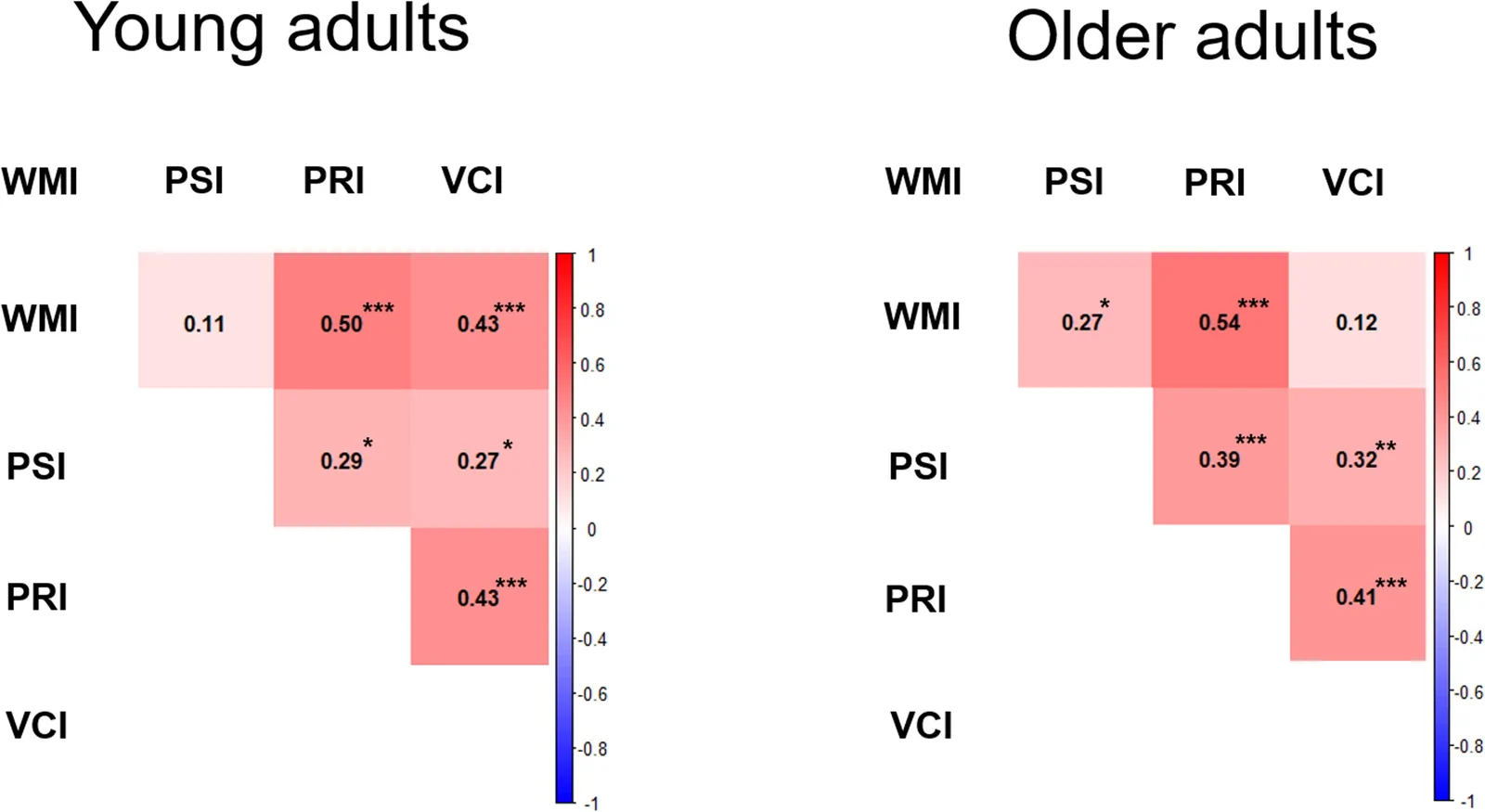
Heat maps for correlations among WAIS-IV indices for young and older adults. *Note.* Working memory index (WMI); Processing speed index (PSI), Perceptual reasoning index (PRI); Verbal comprehension index (VCI). * *p* < .05; ** *p* < .01; *** *p* < .001

We tested the degree of inter-relatedness between each WAIS-IV indices in our sample. Figure 3 depicts the correlation between each pair of cognitive abilities. The qualitative pattern of relationships among variables was relatively similar across age groups. For both age groups, there were positive relationships among all the indices compared. Thus, these results indicate that inter-related structure of cognitive abilities is preserved across different age groups.

### Cognitive abilities and categorization

**Fig. 4:**
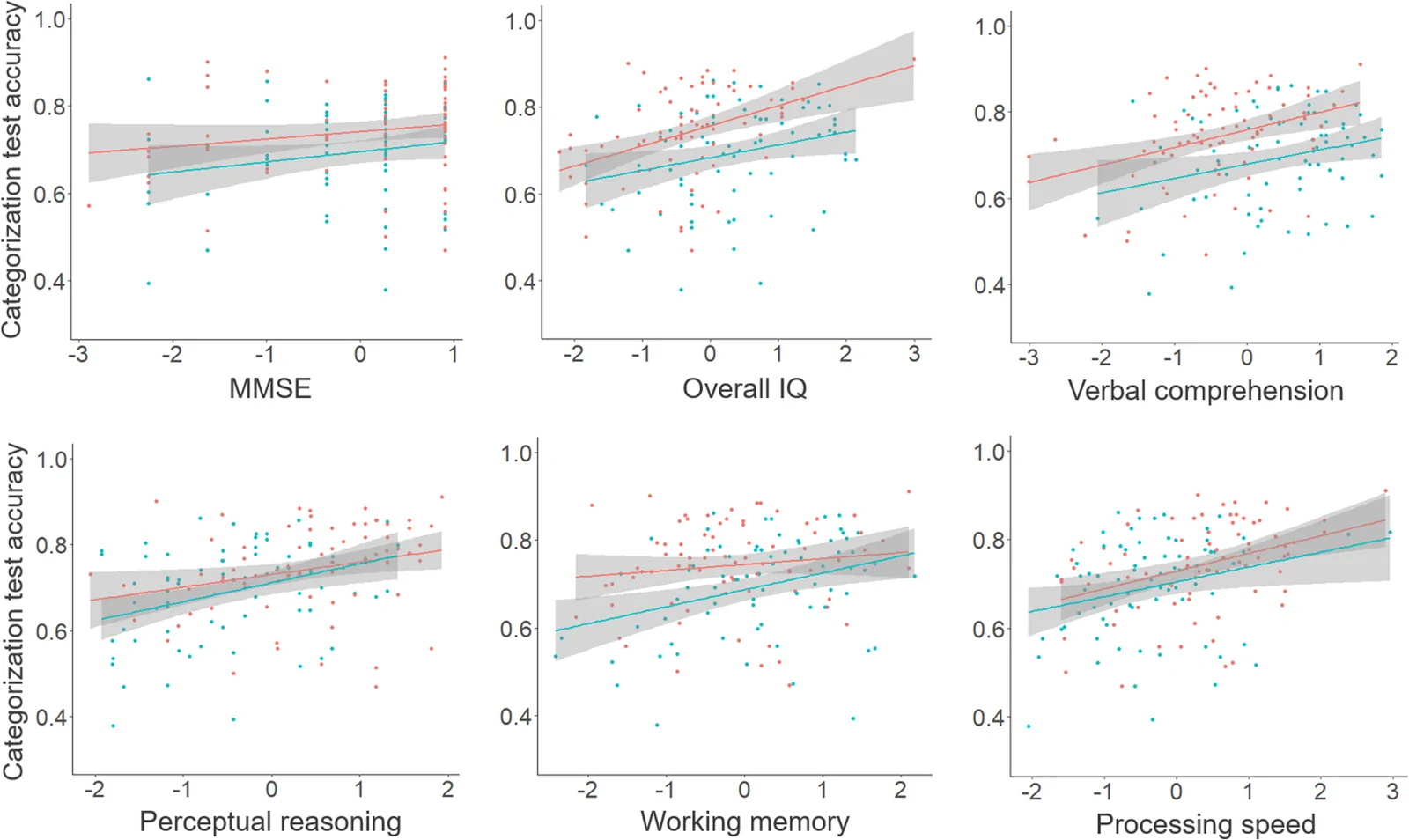
Categorization test accuracy predicted by individual differences in cognitive abilities. *Note.* Each measure was z-scored prior to the plotting. Points represent individual participants. Colors indicate age groups (young adults – red; older adults – blue). The line shows the best-fitting linear regression with the shaded bands representing 95% confidence intervals

Since category learning is thought to involve multiple abilities, we investigated whether any of the WAIS indices predicted categorization test accuracy, and whether there was any cognitive ability that was particularly relevant for maintaining categorization test accuracy in older age. Because categorization test accuracy was moderately correlated across sessions for both young (*r*(74) = 0.30, *p* = .009) and older adults (*r*(71) = 0.40, *p* < .001), and because there was only one measure of each cognitive variable included, we averaged across sessions in computing the dependent score for these regression models. We note, however, that the pattern of results remained the same when we conducted analyses separately for session 1 and session 2. Our first approach was to conduct a separate regression for each cognitive variable (total MMSE score, WAIS full-scale IQ, and the four age-uncorrected WAIS indices; Fig. 4). We applied a Bonferroni correction for the 6 models conducted (corrected alpha = 0.008). Cognitive health as measured by the MMSE (*β* = 0.02, *t* = 2.25, *p* = .03), was not significantly associated with categorization test accuracy following correction for multiple comparisons, and this relationship was not moderated by age group (*ß* = 0.01, *t* = 0.36, *p* = .72). The WAIS full-scale IQ (Overall IQ, *β* = 0.05, *t* = 4.27, *p <* .001), verbal comprehension abilities (*β* = 0.04, *t* = 3.93, *p* < .001), perceptual reasoning abilities (*β* = 0.04, *t* = 3.98, *p* < .001), working memory (*β* = 0.03, *t* = 2.99, *p* = .003), and processing speed (*β* = 0.04, *t* = 4.08, *p* < .001) were each significant positive predictors of categorization test accuracy across age groups, without significant age moderation (all *|ß’s*| < 0.03, |*t*’s| < 1.44, *p*’s > 0.15).

We also entered each age-uncorrected index from the WAIS into one multiple regression model to test whether any one cognitive ability was a better predictor of categorization while accounting for other predictors (Table 5). Prior to computing the multiple regression, we confirmed that all VIFs were within an acceptable range (all VIFs < 4). The overall model was significant, *F*(9,139) = 5.08, *R*^*2*^ = 0.20, *p* < .001. Age group, verbal comprehension, and processing speed were significant predictors of categorization test accuracy, while accounting for other indices. None of the age interaction effects reached significance. Across age groups, better verbal comprehension and faster processing speed were associated with higher categorization test accuracy. Yet, these individual differences did not fully account for age differences in categorization as the age group effect remained significant. Taken together, consistent with the multidimensional nature of categorization, there was a general trend for better cognitive abilities to be associated with higher categorization test accuracy. Verbal comprehension and processing speed stood above the others as especially related to categorization, and the relationships between cognitive abilities and categorization test accuracy were relatively age invariant.

**Table 5:** Linear regression model predicting categorization accuracy from cognitive abilities

Predictors	β (SE)	t	*p*
Age group	− 0.04(0.02)	-2.00	0.048*
VCI	0.02(0.01)	2.36	0.02*
PRI	0.01(0.01)	1.24	0.22
WMI	0.01(0.01)	0.88	0.38
PSI	0.02(0.01)	2.30	0.02*
VCI x age group	− 0.01(0.01)	− 0.63	0.53
PRI x age group	0.01(0.02)	0.32	0.75
WMI x age group	0.03(0.02)	1.61	0.11
PSI x age group	− 0.02(0.02)	− 0.87	0.39

Note. Age-uncorrected scores were used as predictors. *p < .05

### Stability and intercorrelation of state of mind factors

**Fig. 5:**
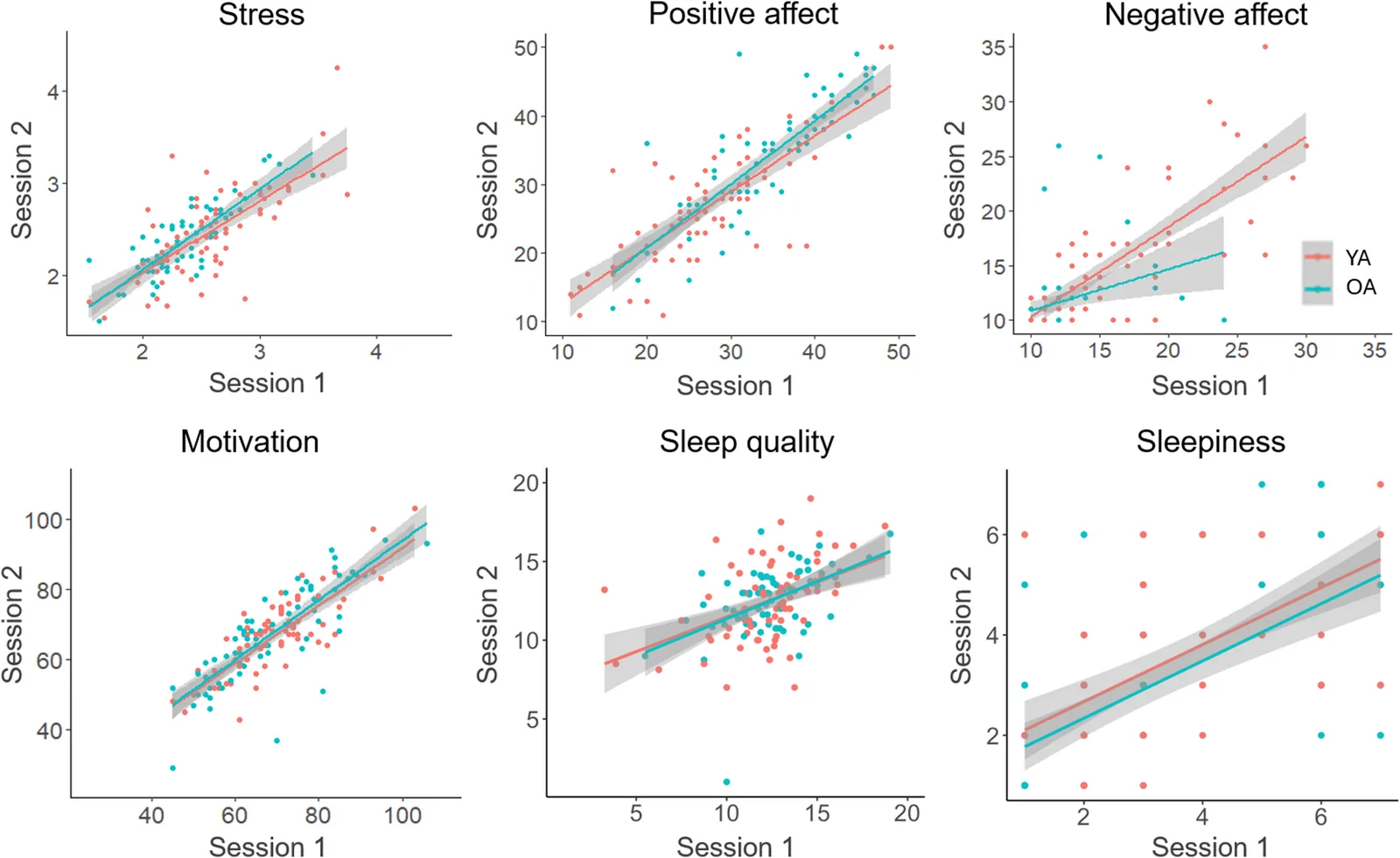
State of mind variables in session 1 vs. session 2. *Note.* Points represent individual participants. Colors indicate age groups (young adults – red; older adults – blue). The line shows the best-fitting linear regression with the shaded bands representing 95% confidence intervals

Figure 5 depicts the stability of state of mind variables across the two sessions. Session 1 scores were significantly correlated with session 2 scores for each state of mind variable in both age groups (all *r’s* > 0.32, *t*’s > 2.85, *p’s* < 0.006). Thus, while each of these constructs has the potential to vary day-to-day, we found relative stability across the two timepoints measured.

**Fig. 6:**
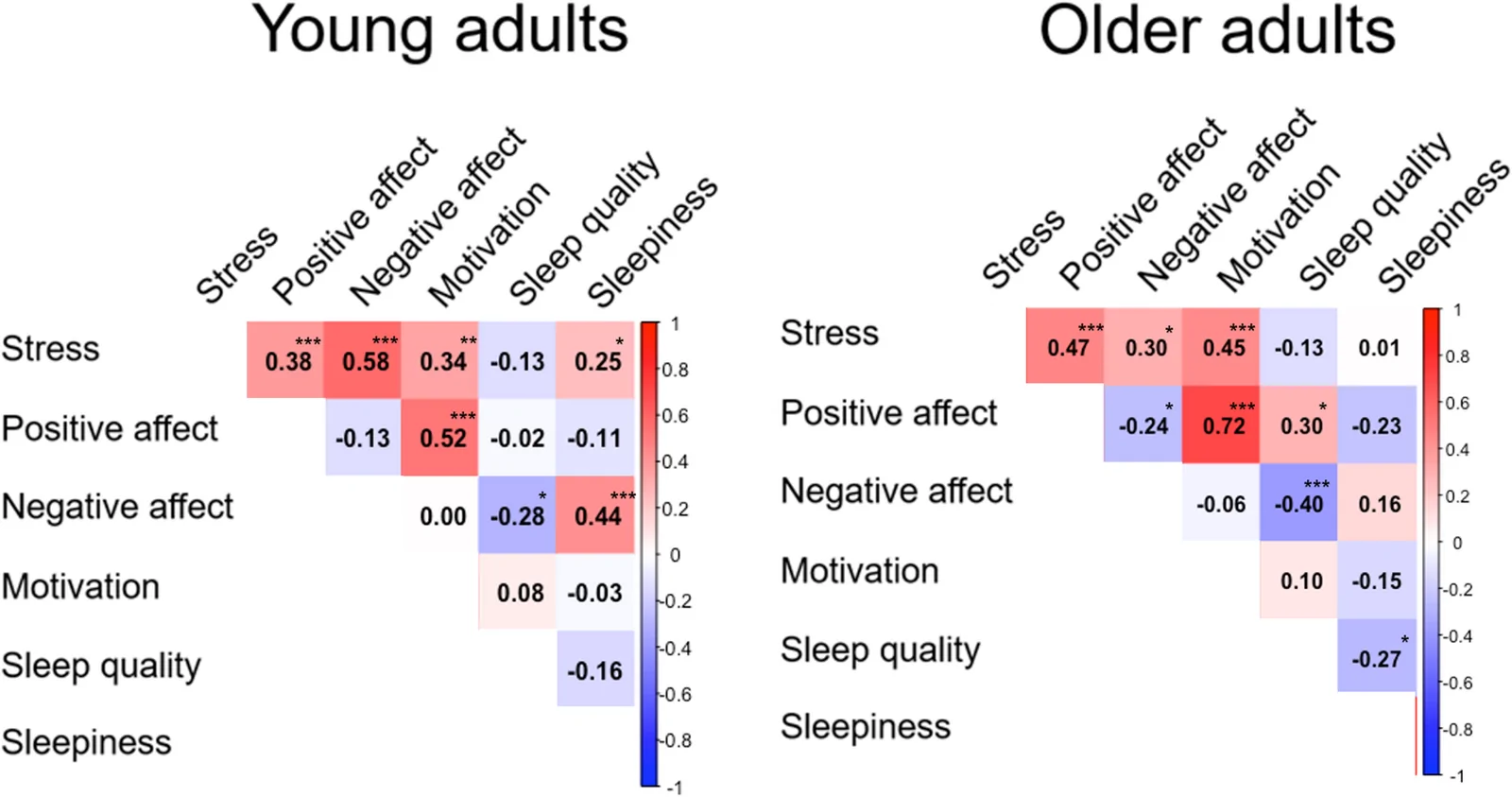
Heat maps for correlations among state of mind variables for young and older adults. *Note*. * *p* < .05; ** *p* < .01; *** *p* < .001

While each of the state of mind factors that we considered are often studied as separate constructs, they can be linked to one another, such as higher levels of positive affect being associated with higher motivation (Erez & Isen, 2002; Isen & Reeve, 2005). Thus, we wanted to determine the degree of inter-relatedness between stress, mood, motivation, and sleep in our sample. Figure 6 depicts the correlation between each pair of state of mind variables. The qualitative pattern of relationships among variables was relatively similar across age groups. For both age groups, there were positive relationships among stress, affect, and motivation, likely reflecting individuals’ overall level of engagement, alertness, and activation. Poorer sleep quality was associated with greater sleepiness (significantly in older adults, but only numerically in young adults), and indicators of poor sleep were associated with greater negative affect in both age groups. Thus, there was also a constellation of variables marking negative mood and lack of alertness reflected in the state of mind data.

### State of mind and categorization accuracy

**Fig. 7:**
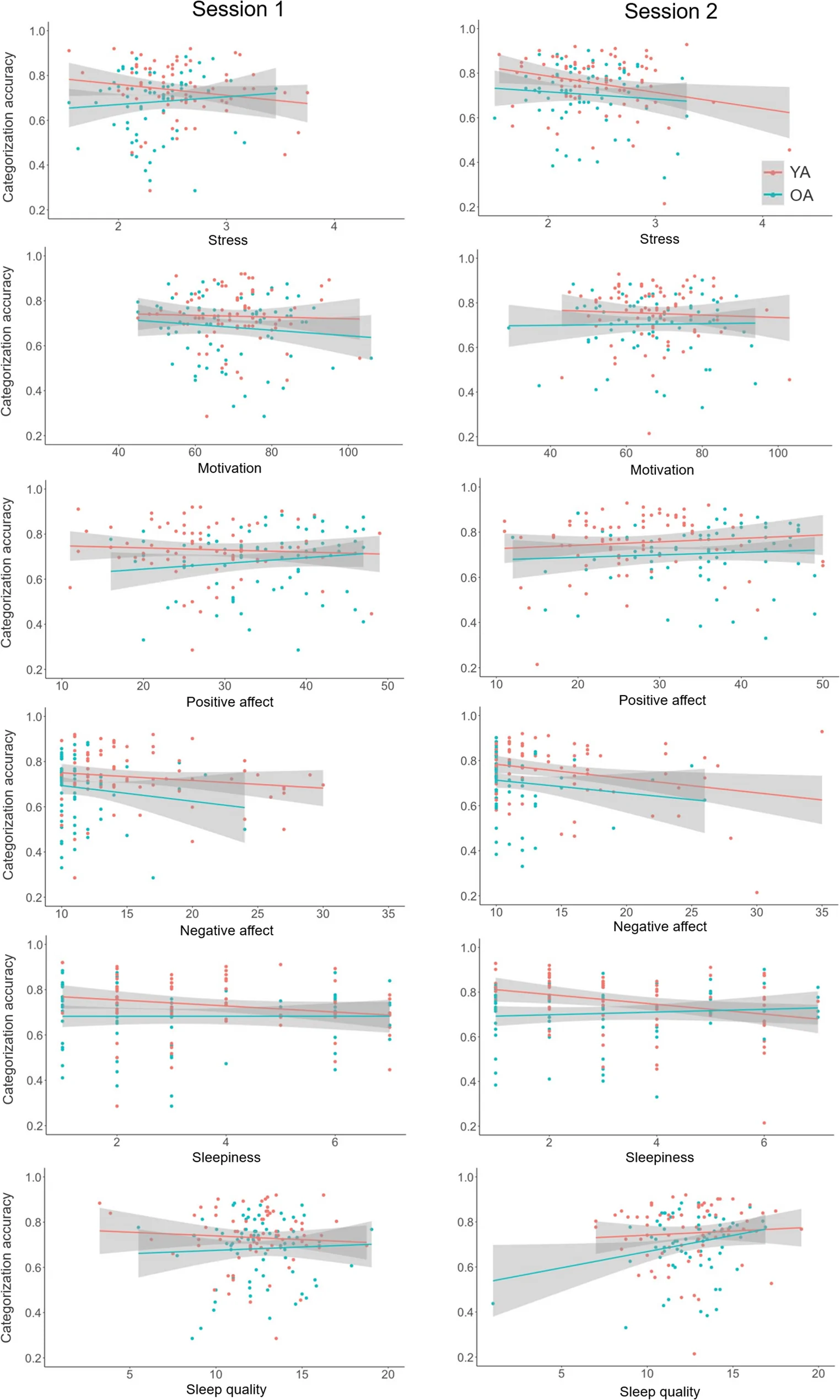
Categorization test accuracy predicted by state of mind variables. *Note.* Points represent individual participants. Colors indicate age groups (young adults – red; older adults – blue). The line shows the best-fitting linear regression with the shaded bands representing 95% confidence intervals

Figure 7 shows the relationship between categorization and state of mind for each session separately. We used linear mixed-effects models to measure the relationship between each state of mind variable and categorization test accuracy, including each session as a separate timepoint. Results of these linear mixed-effects models are displayed in Table 6. After Bonferroni correction for 4 separate models (alpha = 0.0125), age group was a significant predictor for categorization test accuracy in all the models, indicating that no state of mind variable could fully account for age differences in categorization test accuracy. There was also a significant age group and negative affect interaction such that young adults’ categorization test accuracy was more affected by current negative emotions than older adults’ categorization test accuracy. Bayesian model comparisons consistently favored the null model. The stress (BF_01_ = 9.0) and motivation (BF_01_ = 6.67) models showed moderate evidence for null results. The sleep model (BF_01_ = 1.35) indicated anecdotal evidence for the absence of an association between sleep-related variables and categorization test accuracy, consistent with these effects having passed a conventional alpha = 0.05 but not a corrected alpha. Similar to findings from negative affect, there were some signs that young adults were more susceptible to the negative effects of current sleepiness and poor sleep quality. Taken together, state of mind fluctuations had a limited contribution to categorization test accuracy, with the exception that more negative affect was associated with poorer categorization test accuracy, especially in young adults.

**Table 6:** Linear mixed-effects model predicting accuracy by state of mind variables

Predictors	β (SE)	t	*p*	95% CI	
			(Uncorrected)	LL	UL
*Stress model*	Marginal/Conditional *R*^*2*^ = 0.03/0.60, *p* < .001				
Stress	− 0.01(0.01)	− 0.70	0.48	− 0.02	0.01
Age group	− 0.05(0.02)	-2.61	0.010*	− 0.08	− 0.01
Stress x age	0.01(0.02)	0.63	0.53	− 0.02	0.04
*Motivation model*	Marginal/Conditional *R*^*2*^ = 0.04/0.60, *p* < .001				
Motivation	− 0.01(0.01)	-1.35	0.18	− 0.02	0.004
Age group	− 0.05(0.02)	-2.55	0.0118*	− 0.08	− 0.01
Motivation x age	0.001(0.01)	0.07	0.94	− 0.03	0.03
*Affect model*	Marginal *R*^*2*^/Conditional *R*^*2*^ = 0.08/0.62, *p* < .001				
Positive affect (PA)	0.01(0.01)	0.96	0.34	− 0.01	0.02
Negative affect (NA)	− 0.01(0.01)	-1.50	0.13	− 0.03	0.003
Age group	− 0.06(0.02)	-3.13	0.002*	− 0.10	− 0.02
PA x age	0.02(0.02)	0.94	0.35	− 0.02	0.05
NA x age	0.05(0.01)	3.26	0.001*	0.02	0.08
*Sleep model*	Marginal *R*^*2*^/Conditional *R*^*2*^ = 0.05/0.60, *p* < .001				
Sleepiness	− 0.01(0.01)	-1.52	0.13	− 0.02	0.003
Sleep quality	0.01(0.01)	0.96	0.34	− 0.01	0.02
Age group	− 0.05(0.02)	-2.77	0.006*	− 0.08	− 0.01
Sleepiness x age	0.03(0.01)	2.03	0.04	0.001	0.05
Sleep quality x age	0.03(0.01)	2.27	0.02	0.003	0.05

Note. Each variable was z-scored prior to the model fit. Four different models, all with random intercept and fixed mean. Overall model fit was calculated by comparing each model to null model. *p < Bonferroni-corrected alpha = 0.0125

## Discussion

In the present study, we sought to understand how a range of cognitive abilities and state of mind factors related to the ability to learn new categories and the extent to which those relationships differ in older age. We found that categorization test accuracy was relatively stable from one session to another for both young and older adults, and that individual differences in categorization test accuracy were related to overall cognitive abilities without strong age-related moderation. Across age groups, better verbal comprehension, better perceptual reasoning abilities, better working memory, and faster processing speed were associated with better categorization test accuracy, and that verbal abilities and processing speed predicted categorization test performance above-and-beyond the other abilities. We also found that the state of mind factors were not reliably related to categorization test accuracy, except that young adults performed worse on categorization test with higher negative emotions while older adults seem to be relatively unaffected.

Our findings showed that both young and older adults have similar categorization test accuracy across two sessions, providing new evidence that categorization abilities are relatively stable in both age groups. Although the stimuli were different across the two sessions, the category structure was constant. Future work will be needed to test whether individual differences in categorization test accuracy are stable when the categories tested vary more substantially. Further, accuracy on training items versus generalization items are typically separated when computing comparisons of mean scores across conditions based on potential differences in their underlying cognitive processes (Ashby & Maddox, 2011). However, these scores were highly correlated at the individual differences level in the present sample, and we were not able to identify separate cognitive correlates of each. Future research should optimize individual differences measures for distinguishing between the ability to remember trained items and the ability to generalize or else collect very large samples, which would allow for identifying unique cognitive correlates of each while controlling for the other.

The multifaceted nature of categorization was reflected in our results. Overall IQ – which combines across verbal knowledge, reasoning, processing speed, and working memory abilities – was a significant predictor for categorization test accuracy in both young and older adults without significant age moderation. This finding is consistent with the wide range of cognitive abilities that have been linked to categorization (Lewandowsky et al., 2002; Markman & Ross, 2003; Rehder & Hoffman, 2005; Shepard et al., 1961; Zeithamova & Maddox, 2007). While working memory (Lewandowsky, 2011; Lewandowsky et al., 2012) and perceptual reasoning (Jacoby et al., 2022) have important contribution to categorization abilities, it does not seem to uniquely determine who can learn category in an age heterogenous sample. This relationship between overall IQ and categorization test accuracy also raises the possibility that a short categorization test could function as a domain-general indicator of cognitive efficiency. The moderate correlation between categorization test accuracy and WAIS-IV composites suggest that categorization may reflect broad processing demands such as integrating perceptual information and updating decisions that go beyond traditional cognitive domains. A short categorization test could serve as a rapid screening tool that provides an initial estimate of general cognitive functioning, similar to the recent work validating ultra-brief cognitive assessment (Schubert et al., 2024). Such a tool may not substitute for detailed assessment of verbal and reasoning abilities, but it may offer a faster method for identifying individuals who may benefit from more comprehensive testing. We did not, however, find a significant relationship between categorization test accuracy and MMSE scores, another broad measure of cognitive abilities. Yet the MMSE is intended to distinguish between healthy and pathological cognitive states (Folstein et al., 1975), and our sample only included cognitively healthy participants. Future research will be needed to test the feasibility of using categorization test accuracy as a quick indicator of cognitive abilities within a healthy sample and as an indicator of pathological cognitive states in a broader sample.

Beyond overall IQ, we also found a positive relationship between verbal comprehension and categorization test accuracy in both young and older adults while controlling for other WAIS indices. Existing literature has shown that there is a positive relationship between verbal abilities and categorization (Lupyan et al., 2007). Our study extended the investigation of the relationship between verbal abilities and categorization to older adults, revealing that, like adolescents and young adults, higher levels of conceptual knowledge of vocabulary and other world knowledge help older adults perform better in categorization tasks – even when the to-be-learned categories are lab-generated and not existing, real-world categories. This finding has potential implications for theories of concept learning. One possibility is that individuals with higher verbal abilities have a longer history of effective concept learning, such that their verbal abilities are the cumulative product of repeated success in acquiring and refining abstract knowledge. Alternatively, better conceptual knowledge may facilitate the integration of newly encountered concepts by mapping the novel information onto existing semantic frameworks. These two interpretations are not mutually exclusive. Verbal abilities may simultaneously reflect a long history of successful concept acquisition and serve as an active mechanism that facilitates the integration of new concepts. Individuals with richer semantic networks may therefore benefit both from the cumulative effects of prior learning and from more efficient mapping of novel information onto existing knowledge structures. Further studies will be necessary to disentangle these real-time contributions to concept learning to establish whether one or both of these mechanisms drive concept learning abilities.

Faster processing speed was also a predictor for better categorization test accuracy above and beyond other predictors. Though the literature in processing speed in relation to categorization is limited, a recent study found that older adults who are slower in processing performed worse in categorization tasks than young adults with slower processing speed did (Camblats et al., 2022). Our study, however, did not find the interaction effect, but both young and older adults showed a positive relationship between processing speed and categorization test accuracy. This could be explained by the self-paced design of our categorization test, while other studies often have a time limit in their cognitive tasks. Thus, age differences in processing speed may only affect older adults’ categorization test accuracy disproportionately in the context of time pressure while showing a comparable benefit of faster processing speed across ages when there are no time constraints. One caveat, however, is that the training phase in our task *was* time-limited, albeit with a relatively long (4 s) period to respond. While responses were generally well within this response period (mean = 1.34, 95% CI [1.33, 1.35]), it is possible that this constraint did disproportionately affect older adults with slow processing speed, but that this effect was masked by other differences in our design compared to the work by Camblats and colleagues (2022). Future work manipulating the timing of the training and testing phases will be necessary to better understand the processing speed-categorization accuracy relationship in older age.

Regarding state of mind, we found that these predictors were not significantly associated with categorization test accuracy across age groups except for negative affect. There was anecdotal to moderate evidence in favor of the null models for these non-significant relationships. The absence of meaningful correlations between the categorization task and self-reported state of mind in the present study mirrors an existing literature showing that behavioral task performance often fails to align with self-reported abilities or traits (Dang et al., 2020). Similar dissociations have been noted in the category learning literature, where behavioral performance and subjective reports frequently diverge (Ashby & Maddox, 2011). Thus, present null finding should not be interpreted as anomalous, but rather as further evidence that behavioral and self-report measures may index partially distinct processes.

Our results, however, suggest that young adults appeared to be more influenced by negative affect than older adults in our categorization task. This pattern is generally consistent with prior research suggesting that older adults show a “positivity effect” characterized by reduced attention to and the processing of negative emotions compared to young adults (Ashley & Swick, 2009; Ding et al., 2022; Nashiro et al., 2012; Steenhaut et al., 2019). While existing research has demonstrated that affect can influence categorization processes (Ramon et al., 2007), the direct impact of negative affect on categorization accuracy was still underexplored. The present finding, however, should be interpreted with caution as our sample of older adults reported significantly lower levels of negative affect overall compared to young adults. As a result, the observed effect appears to be driven primarily by the young adult group. This makes it difficult to determine whether the difference reflects a true increase in susceptibility among young adults, a reduced baseline of negative affect among older adults, or a combination of both factors.

Overall, stable cognitive traits such as overall IQ appear to have a clearer influence on categorization test accuracy than transient state of mind variables. Research in theories of cognitive reserve emphasize that accumulated knowledge provides a stable foundation for test accuracy, even when individuals experience fluctuations in daily mood or fatigue (McDonough et al., 2022; Oosterhuis et al., 2023). In contrast, while acute stress or sleepiness may temporarily modulate attention or flexibility, these effects are often inconsistent and less predictive once stable traits are accounted for (Hur et al., 2015; McDonough et al., 2022). This means that interventions aimed at supporting concept learning and categorization in later life may benefit from working with individual’s existing stable traits rather than attempting to directly manipulate transient states like momentary stress or motivation. However, we note that evidence for cognitive training is limited, with studies indicating that individuals learn task-specific strategies rather than broad changes in underlying cognitive abilities (Melby-Lervåg et al., 2016; Watrin et al., 2022).

While the present study revealed the null relationships between stress, motivation, sleep and categorization test accuracy in both young and older adults, more within subject datapoints are needed to determine the pattern and magnitude of the effects of state of mind on categorization. While each of the factors we considered were conceptually aspects of state of mind, having only two measurement occasions meant that we could not fully take advantage of multilevel modeling techniques to assess intraindividual variability (Vanderhasselt et al., 2014; Almeida et al., 2009; Smith et al., 2004). Thus, we may have missed the true ‘state’ effects of state of mind that are only present within individuals relative to their own baseline. Yet we also saw strong, stable interindividual differences in our state of mind variables as evidenced by the correlation across subjects for the two sessions. Additionally, our sample was predominantly Caucasian and highly educated. 79% of our full sample was non-Hispanic white, compared to 58% in general population (U.S. Census Bureau, 2020). Thus, out sample is not fully representative of the U.S. population and could limit the generalizability of the findings. While achieving full representation with a small sample is challenging, working towards a more balanced sample would improve confidence in the generalizability of the findings.

In sum, results of the present study showed that higher IQ, better verbal abilities and faster processing speed are associated with better categorization test accuracy in both young and older adults, suggesting that various cognitive abilities are associated with categorization abilities regardless of age group. Although stable cognitive abilities may be positively correlated with categorization test accuracy, transient state of mind variables were not associated with categorization test accuracy except for negative affect. Therefore, it is important to establish educational and cognitive interventions to emphasize verbal and conceptual knowledge as it makes category learning more efficient even in older age. Though slow processing speed is a part of natural aging process, it may be helpful to emphasize the training of processing speed in older population such as *Useful Field of View* training paradigm (Edwards et al., 2018) to optimize category learning.

## Data Availability

The authors affirm that all data, analytic methods, and study materials associated with this research will be made publicly available to other researchers via the Open Science Framework (OSF) to facilitate transparency and reproducibility. Interested parties will be able to access these resources at [https://osf.io/gsn47/](https:/osf.io/gsn47) . This study was not pre-registered.

## Declarations

### Public significance statements

The ability to learn new concepts is critical throughout the lifespan, helping us to adjust to an ever-changing world and adopt new skills and hobbies that can enrich our lives. In the present research, we found that cognitive abilities were more predictive of concept learning abilities across age than state of mind factors, with current conceptual knowledge and processing speed being key factors that led to better concept learning.
